# Fluoroquinolone Can Be an Effective Treatment Option for Acute Pyelonephritis When the Minimum Inhibitory Concentration of Levofloxacin for the Causative *Escherichia coli* Is ≤16 mg/L

**DOI:** 10.3390/antibiotics10010037

**Published:** 2021-01-02

**Authors:** Yeonjae Kim, Bongyoung Kim, Seong Heon Wie, Jieun Kim, Moran Ki, Yong Kyun Cho, Seung Kwan Lim, Jin Seo Lee, Ki Tae Kwon, Hyuck Lee, Hee Jin Cheong, Dae Won Park, Seong Yeol Ryu, Moon Hyun Chung, Hyunjoo Pai

**Affiliations:** 1 Center for Infectious Disease, National Medical Center, Seoul 04564, Korea; arckyj@nmc.or.kr; 2 Department of Internal Medicine, College of Medicine, Hanyang University, Seoul 04763, Korea; sobakas@hanyang.ac.kr (B.K.); quidam76@hanyang.ac.kr (J.K.); 3 Division of Infectious Diseases, Department of Internal Medicine, St. Vincent’s Hospital, College of Medicine, The Catholic University of Korea, Seoul 06591, Korea; wiesh@catholic.ac.kr; 4 Department of Cancer Control and Policy, Graduate School of Cancer Science and Policy, National Cancer Center, Goyang 10408, Korea; moranki@ncc.re.kr; 5 Department of Internal Medicine, Medical College, Gacheon University, Incheon 21565, Korea; karmacho@gmail.com; 6 Department of Internal Medicine, Ansung Hospital, Gyeonggi Provincial Medical Center, Ansung 17572, Korea; haveabigheart@gmail.com; 7 Division of Infectious Diseases, Kangdong Sacred Heart Hospital, Hallym University, Seoul 05355, Korea; rem324@naver.com; 8 Department of Internal Medicine, School of Medicine, Kyungpook National University, Daegu 41566, Korea; ktkwon@knu.ac.kr; 9 Division of Infectious Diseases, Dong-A University Hospital, Dong-A University, Busan 49201, Korea; hyucklee@gmail.com; 10 Division of Infectious Diseases, Korea University Guro Hospital, Seoul 08308, Korea; heejinmd@korea.ac.kr; 11 Division of Infectious Diseases, Korea University Ansan Hospital, Ansan 15355, Korea; pugae1@hanmail.net; 12 Division of Infectious Diseases, Dongsan Hosptial, Keimyeong University, Daegu 41931, Korea; 121rsy@hanmail.net; 13 Division of Infectious Diseases, Seogwipo Medical Center, Jeju 63585, Korea; mhchungid@daum.net

**Keywords:** urinary tract infection, pyelonephritis, *E. coli*, fluoroquinolone, treatment

## Abstract

The purpose of this study was to determine whether the fluoroquinolone (FQ) minimum inhibitory concentration (MIC) for the causative agent *Escherichia coli* influences the clinical response of FQ treatment at 72 h in patients with community-acquired acute pyelonephritis (CA-APN). We prospectively collected the clinical data of women with CA-APN from 11 university hospitals from March 2010 to February 2012 as well as *E. coli* isolates from the urine or blood. In total, 78 patients included in this study received FQ during the initial 72 h, and the causative *E. coli* was detected. The clinical response at 72 h was significantly higher in patients with a levofloxacin MIC ≤ 16 mg/L than in those with an MIC > 16 mg/L (70.4% vs. 28.6%, *p* = 0.038). No difference was observed in clinical response at 72 h based on ciprofloxacin MIC. To summarize, FQ can be an effective treatment option for CA-APN when levofloxacin MIC against *E. coli* is ≤16 mg/L.

## 1. Introduction

Acute pyelonephritis (APN) is one of the most common bacterial infections in the general population, particularly among women [1]. Fluoroquinolones (FQs), including ciprofloxacin (CIP) and levofloxacin (LEV), are recommended as first-line empirical antibiotics in domestic and international guidelines [2,3].

FQs are broad-spectrum antimicrobial agents frequently prescribed for complicated urinary tract infections (UTIs), given their high oral bioavailability and urinary excretion rate and low bacterial resistance [4]. However, the resistance of urinary isolates to FQ is increasing. APN related to FQ-resistant (FQ-R) uropathogens is associated with lower early clinical response and longer hospitalization [5]. Nonetheless, some patients treated with CIP or LEV without switching antibiotics show reasonable clinical outcomes regardless of their susceptibility [5,6]. The correlation between in vitro susceptibility to FQ and clinical outcomes in patients with UTIs is indeed controversial [7,8].

This study evaluates whether the minimum inhibitory concentration (MIC) of FQ against the causative agent *Escherichia coli* influences the clinical response of FQ treatment at 72 h in patients with community-acquired APN (CA-APN).

## 2. Material and Methods

A prospective, observational, multi-center cohort study of patients with APN was conducted between March 2010 and February 2012. Eleven acute care hospitals in South Korea participated in the study, 10 of which were academic hospitals. The study protocol was approved by the Institutional Review Board (IRB) of Hanyang University Hospital (IRB number: HYUH 2010-007). Clinical, laboratory, and microbiological data were collected from each participating hospital using a web-based medical record system. All data were kept confidential, and the IRB waived off the requirement for written informed consent from the patients.

APN was defined as fever (temperature ≥37.8 °C) and presence of at least three of the following conditions: (1) symptoms of lower UTI defined as painful urination, urgency, frequency, or pain in the suprapubic area; (2) flank pain; (3) costovertebral angle (CVA) tenderness; (4) leukocytosis (peripheral white blood cell count ≥20,000/mm^3^ or polymorphonuclear cells ≥65%); and (5) pyuria defined as presence of 10 or more leukocytes per high-power field [9]. CA-APN was defined as a case attending the emergency department or outpatient clinic from the community with signs of APN described above.

Patients with CA-APN were enrolled by infectious disease specialists, and all patients detected with causative pathogens through urine or blood cultures were included in the analysis. Patients diagnosed with APN more than 48 h after admission and those with catheter-associated UTI were excluded. Patients aged <15 years, those who had other reasons for pyuria and fever, or those with insufficient data were also excluded. We collected variables such as demographic characteristics, clinical features, laboratory findings, and clinical outcomes. Charlson comorbidity index and Pitt bacteremia score are defined as previously described [10].

Empirical FQ use was defined as patients receiving FQ for at least 72 h before obtaining microbiology results. Clinical response at 72 h was defined if the following criteria were met at 72 h following inception of empirical antibiotics: (1) resolution of fever and (2) improvement of UTI symptoms or signs [9]. Relapse was defined as recurrence of UTI symptoms within 7–14 days after the completion of therapy.

Urine cultures were positive for pathogen following detection of ≥10^5^ colony-forming unit (CFU)/mL. FQ susceptibility and extended-spectrum β-lactamase (ESBL) positivity were determined with a semi-automated system in each clinical laboratory. The isolates from patients receiving empirical FQ therapy were used to derive the MICs of LEV and CIP by E-test (AB-BIODISK, Solna, Sweden). Resistance was defined according to the criteria of the Clinical and Laboratory Standards Institute (CLSI) [11].

Categorical variables were compared using the chi-square test or Fisher’s exact test. Continuous variables were analyzed by the Mann–Whitney U test or independent *t*-tests. A value of *p* < 0.05 in a two-tailed test was considered statistically significant. Statistical analyses were conducted using SPSS version 24.0 for Windows (IBM Corp., Armonk, NY, USA). 

## 3. Results

In total, 1138 women with CA-APN were screened, of which 390 were excluded due to the absence of results indicating causative pathogens. Among 748 patients with proven causative pathogens along with susceptibility test results, *E. coli* was identified as a causative pathogen for UTI in 686 patients (91.7%). Of 686 patients, 78 patients received empirical FQ for at least 72 h before the identification of causative organisms and were finally included in this study.

The clinical characteristics of the subjects are presented in Table 1. Of these patients, 36.1% had a history of antibiotic use within 1 year and 24.4% had diabetes as an underlying disease. The causative *E. coli* from 17.7% of patients who used empirical FQ was resistant to FQ. Of patients who used empirical FQ, 39.7% changed antibiotics during the hospitalization period. Overall, the clinical response rate after 72 h of FQ treatment was 66.7% (52/78); mortality and relapse rates were 2.6% and 3.8%, respectively. The median hospitalization duration was 7 days.

**Figure 1:**
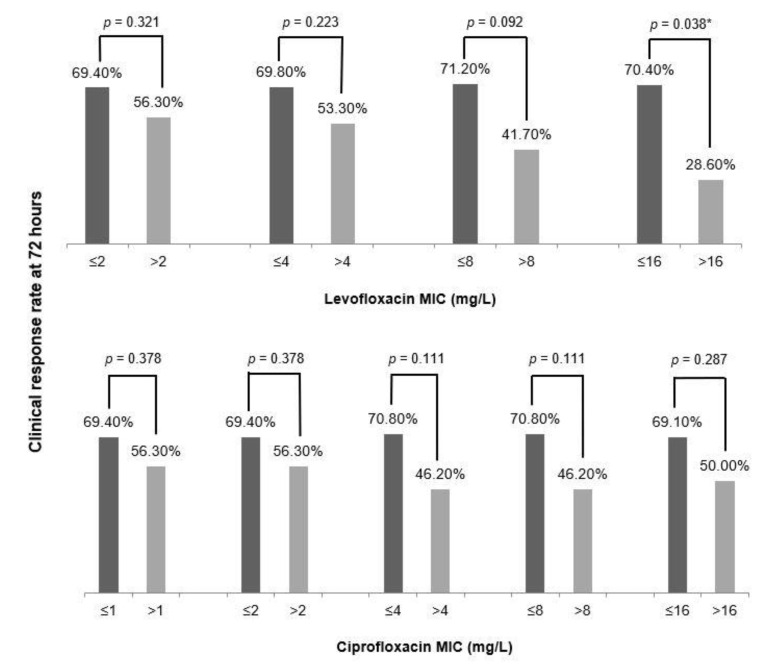
Correlation between minimal inhibitory concentrations (MICs) of levofloxacin and ciprofloxacin and clinical response rates at 72 h after the inception of empirical fluoroquinolone treatment in patients with acute pyelonephritis. * *p* < 0.05.

The correlation between LEV and CIP MICs against the causative *E. coli* and clinical response at 72 h of FQ treatment is presented in Figure 1. Considering the MIC of LEV, the clinical response rate at 72 h of FQ treatment significantly differed between patients with an LEV MIC ≤ 16 mg/L and those with an LEV MIC > 16 mg/L (70.4% vs. 28.6%, *p* = 0.038). For CIP, no significant difference was observed in the clinical response rate at 72 h based on the MICs of isolates (70.1% vs. 45.5%, *p* = 0.165). Further, no significant differences were noted in the clinical characteristics of patients with an LEV MIC ≤ 16 mg/L and those with an LEV MIC >16 mg/L as well as patients with a CIP MIC ≤ 16 mg/L and those with a CIP MIC > 16 mg/L (Table S1).

## 4. Discussion

FQs are one of the most widely used antimicrobial agents in the clinical setting, but there has been growing concerns about their utility owing to an increase in bacterial resistance [4,12,13]. Although previous studies have revealed the association of inadequate empirical treatment of *E. coli* bacteremia with increased mortality, the bacteremia related to UTI mostly causes little mortality and the urinary concentration of FQ is high [14]. Hence, the standard use of FQs against urinary infections by FQ-R pathogens needs to be re-evaluated. Several studies have suggested the applicability of FQs even in APN patients with FQ-R pathogens [5,15,16]. The case of a young female with an uncomplicated UTI caused by CIP-resistant *E. coli* (MIC > 4 mg/L) was improved following FQ administration [16], and three out of four APN patients infected with FQ-R *E. coli* were successfully treated with LEV or CIP [15]. In another study, FQ treatment without any change in antibiotic regimen provided reasonably good clinical outcomes in patients with FQ-R pathogens [8]. High urine concentrations and prolonged bactericidal effects of CIP and LEV could be the possible reasons for clinical success in such cases [17,18].

According to the CLSI and European Committee on Antimicrobial Susceptibility Testing (EUCAST) standards, breakpoints of norfloxacin for uncomplicated UTI exist; however, values for LEV and CIP in UTIs have not been determined [19,20]. In a pharmacokinetic and pharmacodynamic model of high-dose LEV (750 mg/day), clinical failure was predicted when the MIC of LEV was >32 mg/L [8]. Indeed, in our study, we observed a good clinical response after 72 h of FQ use in patients infected by pathogens with an LEV MIC ≤ 16 mg/L. Therefore, we suggest that the MIC breakpoints of LEV for UTIs need to be increased above the general standard breakpoints.

This study has some potential limitations. First, the sample size of the study was relatively small. According to post-hoc power analysis using G*power version 3.1 (Düsseldorf University, Germany), the power was calculated as 0.74 with our sample size (*n* = 78). Considering the nature of the study design and complete observation within the study cohort, the power might not be enough but could be a tolerable level. Second, our study was mainly conducted in large medical centers, and many of the patients had underlying comorbidities. Therefore, the patients enrolled in the study may not represent the entire population of patients with CA-APN. Third, our definition of causative pathogen might be controversial. In fact, there were two participants for whom *E. coli* was detected only through blood cultures, not through urine cultures. Despite the fact that the portal of entry of *E. coli* can be controversial, we included those cases because their clinical symptoms and signs were compatible to APN. We weigh the possibility of improper handling of urine samples more than that of the existence of other infectious diseases. Finally, the isolates studied herein were collected from 2010 to 2012 and may not accurately reflect the current situation.

## 5. Conclusions

In conclusion, FQ treatment showed a good clinical response at 72 h in CA-APN when the LEV MIC against the causative *E. coli* isolates was ≤ 16 mg/L.

## Figures and Tables

**Table 1:** Clinical characteristics and outcomes of the women with community-acquired acute pyelonephritis who used fluoroquinolone as empirical therapy.

		Total = 78
Demographic data		
Age, years, mean ± *SD*		58.9 ± 16.5
Past history (%)		
History of antibiotic use within 1 year		22/61 (36.1)
History of urinary tract infection		18/61 (29.5)
History of admission within 1 year		18/69 (26.1)
Co-morbidity condition (%)		
Charlson comorbidity index ≥ 2		15 (19.2)
Diabetes mellitus		19 (24.4)
Cerebrovascular disorder		4 (5.1)
Congestive heart failure		4 (5.1)
Chronic pulmonary disease		2 (2.6)
Chronic liver disease		7 (9.0)
Clinical features (%)		
Flank pain		23 (29.5)
Lower urinary tract infection symptoms ^a^		50 (64.1)
Costovertebral angle tenderness		47 (60.3)
Pitt bacteremia score ≥ 1 ^b^		34 (43.6)
Laboratory findings at presentation (%)		
C-reactive protein > 20 mg/dL		43 (55.1)
White blood cells ≥ 20,000/mm^3^		10 (12.8)
Hematuria (≥5–9 red blood cells/high-power field)		50 (64.1)
Azotemia ^c^		15 (19.2)
ESBL positivity		5 (6.4)
FQ resistance		33 (17.7)
Antibiotic change during hospitalization period (%)		31 (39.7)
Clinical outcomes		
Clinical response after 72 h (%)		52 (66.7)
Overall mortality (%)		2 (2.6)
Overall relapse (%)		3 (3.8)
Hospitalization duration, days (IQR)		7 (5-9)

Abbreviations: *SD*, standard deviation; ESBL, extended-spectrum β-lactamase; FQ, fluoroquinolone; IQR, interquartile range. ^a^ Symptoms of lower urinary tract infection are dysuria, frequency, urgency, and nocturia. ^b^ See reference [10] of Pitt bacteremia score. ^c^ Azotemia was defined as blood urea nitrogen ≥ 20 mg/dL or serum creatinine ≥ 1.4 mg/dL.

## Data Availability

The data presented in this study are available on request from the corresponding author.

## Supplementary Materials

The following are available online at https://www.mdpi.com/2079-6382/10/1/37/s1: Table S1. Comparison of clinical characteristics and outcomes of women with community-acquired acute pyelonephritis who used fluoroquinolone as empirical therapy according to levofloxacin or ciprofloxacin minimum inhibitory concentration of the causative *E. coli*.
