# Supplementary material for: Fluoroquinolone Can Be an Effective Treatment Option for Acute Pyelonephritis When the Minimum Inhibitory Concentration of Levofloxacin for the Causative Escherichia coli Is ≤16 mg/L
Source: Antibiotics (Basel). 2021 Jan 2;10(1):37. doi: 10.3390/antibiotics10010037 (PMC7823373; doi:10.3390/antibiotics10010037)
Supplement: Supplementary file 1 [file antibiotics-10-00037-s001.pdf]

Table S1. Comparison of clinical characteristics and outcomes of women with community-acquired acute pyelonephritis who used fluoroquinolone as empirical therapy according to levofloxacin or ciprofloxacin minimum inhibitory concentration of the causative *E. coli*

	Levofloxacin MIC			Ciprofloxacin MIC		
	≤16mg/L (n=71)	>16mg/L (n=7)	*P*	≤16mg/L (n=67)	>16mg/L (n=11)	*P*
Age, years, mean ±SD	58.8±17.50	60.4±10.9	0.799	58.5±17.4	61.1±10.0	0.495
Past history (%)						
History of antibiotic use within 1 year	21/58 (36.2)	1/3 (33.3)	1.000	20/56 (35.7)	2/5 (40.0)	1.000
History of urinary tract infection	17/56 (30.4)	1/5 (20.0)	1.000	16/53 (30.2)	2/8 (25.0)	0.764
History of admission within 1 year	16/63 (25.4)	2/6 (33.3)	0.647	15/60 (25.0)	3/9 (33.3)	0.687
Co-morbidity condition (%)						
Charlson comorbidity index ≥2	13 (18.3)	2 (28.6)	0.614	12 (17.9)	3 (27.3)	0.434
Diabetes mellitus	16 (22.5)	3 (42.9)	0.352	15 (22.4)	4 (36.4)	0.448
Cerebrovascular disorder	3 (4.2)	1 (14.3)	0.319	3 (4.5)	1 (9.1)	0.463
Congestive heart failure	3 (4.2)	1 (14.3)	0.319	3 (4.5)	1 (9.1)	0.463
Chronic pulmonary disease	2 (2.8)	0 (0)	1.000	2 (3.0)	0 (0)	1.000
Chronic liver disease	7 (9.9)	0 (0)	1.000	6 (9.0)	1 (9.1)	1.000
Clinical features (%)						
Flank pain	20 (28.2)	3 (42.9)	0.414	19 (28.4)	4 (36.4)	0.723
Lower urinary tract infection symptoms[a]	44 (62.0)	6 (85.7)	0.411	40 (59.7)	10 (90.9)	0.086
Costovertebral angle tenderness	41 (57.7)	6 (85.7)	0.233	39 (58.2)	8 (72.7)	0.511
Pitt bacteremia score ≥1[b]	30 (42.3)	4 (57.1)	0.693	30 (44.8)	4 (36.4)	0.747
Laboratory findings at presentation (%)						
C-reactive protein >20 mg/dL	39 (54.9)	4 (57.1)	1.000	37 (55.2)	6 (54.5)	1.000
White blood cells ≥20,000/mm^3	9 (12.7)	1 (14.3)	1.000	9 (13.4)	1 (9.1)	1.000
Hematuria (≥5-9 red blood cells/high-power field)	45 (63.4)	5 (71.4)	1.000	42 (62.7)	8 (72.7)	0.737
Azotemia[C]	15 (21.1)	0 (0)	0.335	15 (22.4)	0 (0)	0.110
Antibiotic change during hospitalization period (%)	26 (36.6)	5 (71.4)	0.107	23 (34.3)	8 (72.7)	0.022
Clinical outcomes (%)						
Clinical response after 72 hours	50 (70.4)	2 (28.6)	0.038	47 (70.1)	5 (45.5)	0.165
Overall mortality (%)	2 (2.8)	0 (0)	1.000	2 (3.0)	0 (0)	1.000
Overall relapse (%)	2 (2.8)	1 (14.3)	0.249	2 (3.0)	1 (9.1)	0.370
Hospitalization duration, days (IQR)	7 (5-9)	11 (5-31)	0.113	7 (5-9)	9 (6-15)	0.055

Abbreviations: MIC, minimum inhibitory concentration; SD, standard deviation; IQR, interquartile range

[a]Symptoms of lower urinary tract infection are dysuria, frequency, urgency, and nocturia

[b]See reference [10] of Pitt bacteremia score

[c]Azotemia was defined as blood urea nitrogen ≥20 mg/dL or serum creatinine ≥1.4 mg/dL
